# Protein body formation in the endoplasmic reticulum as an evolution of storage protein sorting to vacuoles: insights from maize γ-zein

**DOI:** 10.3389/fpls.2014.00331

**Published:** 2014-07-15

**Authors:** Davide Mainieri, Francesca Morandini, Marie Maîtrejean, Andrea Saccani, Emanuela Pedrazzini, Vitale Alessandro

**Affiliations:** Istituto di Biologia e Biotecnologia Agraria, Consiglio Nazionale delle RicercheMilano, Italy

**Keywords:** disulfide bonds, endoplasmic reticulum, evolution of the secretory pathway, seed storage proteins, *Zea mays*

## Abstract

The albumin and globulin seed storage proteins present in all plants accumulate in storage vacuoles. Prolamins, which are the major proteins in cereal seeds and are present only there, instead accumulate within the endoplasmic reticulum (ER) lumen as very large insoluble polymers termed protein bodies. Inter-chain disulfide bonds play a major role in polymerization and insolubility of many prolamins. The N-terminal domain of the maize prolamin 27 kD γ-zein is able to promote protein body formation when fused to other proteins and contains seven cysteine residues involved in inter-chain bonds. We show that progressive substitution of these amino acids with serine residues in full length γ-zein leads to similarly progressive increase in solubility and availability to traffic from the ER along the secretory pathway. Total substitution results in very efficient secretion, whereas the presence of a single cysteine is sufficient to promote partial sorting to the vacuole via a wortmannin-sensitive pathway, similar to the traffic pathway of vacuolar storage proteins. We propose that the mechanism leading to accumulation of prolamins in the ER is a further evolutionary step of the one responsible for accumulation in storage vacuoles.

## INTRODUCTION

Seed storage proteins, the major food proteins, have unique characteristics that allow very high accumulation during seed development, low digestibility by predators, but rapid hydrolysis during germination. Despite these shared features, as well as structures that strongly suggest an evolution through variable combinations of very few domain types (Shewry et al., 1995; Adachi et al., 2001; Xu and Messing, 2008; Gu et al., 2010), the different seed storage proteins have specific assembly, solubility, and intracellular localization properties and can thus be divided into three major classes: (i) 2S albumins (the number refers to the sedimentation constant) are monomeric and soluble in water, (ii) 7S and 11S globulins are homotrimers and homohexamers, respectively, and are soluble in salt water, (iii) prolamins, the most heterogeneous class, are soluble in strong alcohol/water solution or after reduction of disufide bonds. How the evolution of biochemical properties relates to that of subcellular localization is still not clear (Ibl and Stoger, 2012). All storage proteins are co-translationally inserted into the endoplasmic reticulum (ER), where 7S and 11S globulins rapidly trimerize. The ER is the port of entry of the secretory pathway: from here, proteins traffic through the Golgi complex and are secreted by default, unless they have structural features/signals that sort them to intracellular compartments of the pathway. Albumins and globulins follow traffic pathways shared by the other soluble vacuolar proteins and are sorted to storage vacuoles of cotyledonary cells, where they accumulate (Vitale and Hinz, 2005). Endoproteolytic cleavage of 11S globulin polypeptides occurring in the vacuole promotes further assembly of trimers into the final 11S hexamers. Most prolamins, instead, form large polymers that in many cases rapidly become insoluble and do not proceed along the secretory pathway, accumulating as protein bodies (PB) within the ER lumen of endosperm cells (Shewry et al., 1995; Herman, 2008). The developmentally programmed use of the ER as a protein storage compartment seems to be unique to plants, whereas the formation of insoluble protein accretions in the ER is associated to many human diseases (Anelli and Sitia, 2010). Storage albumins and globulins are present throughout land plant evolution, but prolamins have been found only in cereals and are therefore much less ancient (Xu and Messing, 2008). In all cereals, each PB contains many different prolamin polypeptides, indicating promiscuous interactions among the products of the different prolamin genes. When expressed individually in vegetative tissues of transgenic plants, some prolamins form homotypic PBs within the ER, indicating that they contain all the information sufficient to form an ER-retained polymer, whereas others remain soluble and are delivered to the vacuole. Examples of these two different destinies are maize 27 kD γ-zein and wheat γ-gliadin, respectively (Geli et al., 1994; Napier et al., 1997). Individual storage globulins can also be retained in the ER as large accretions, instead of being sorted to storage vacuoles, if the synthesis of other members of the family is suppressed (Kinney et al., 2001). Altogether, these data suggest a close relationship between the mechanisms of storage protein accumulation in vacuoles or the ER, and that the latter may have evolved directly from the former.

**FIGURE 1:**
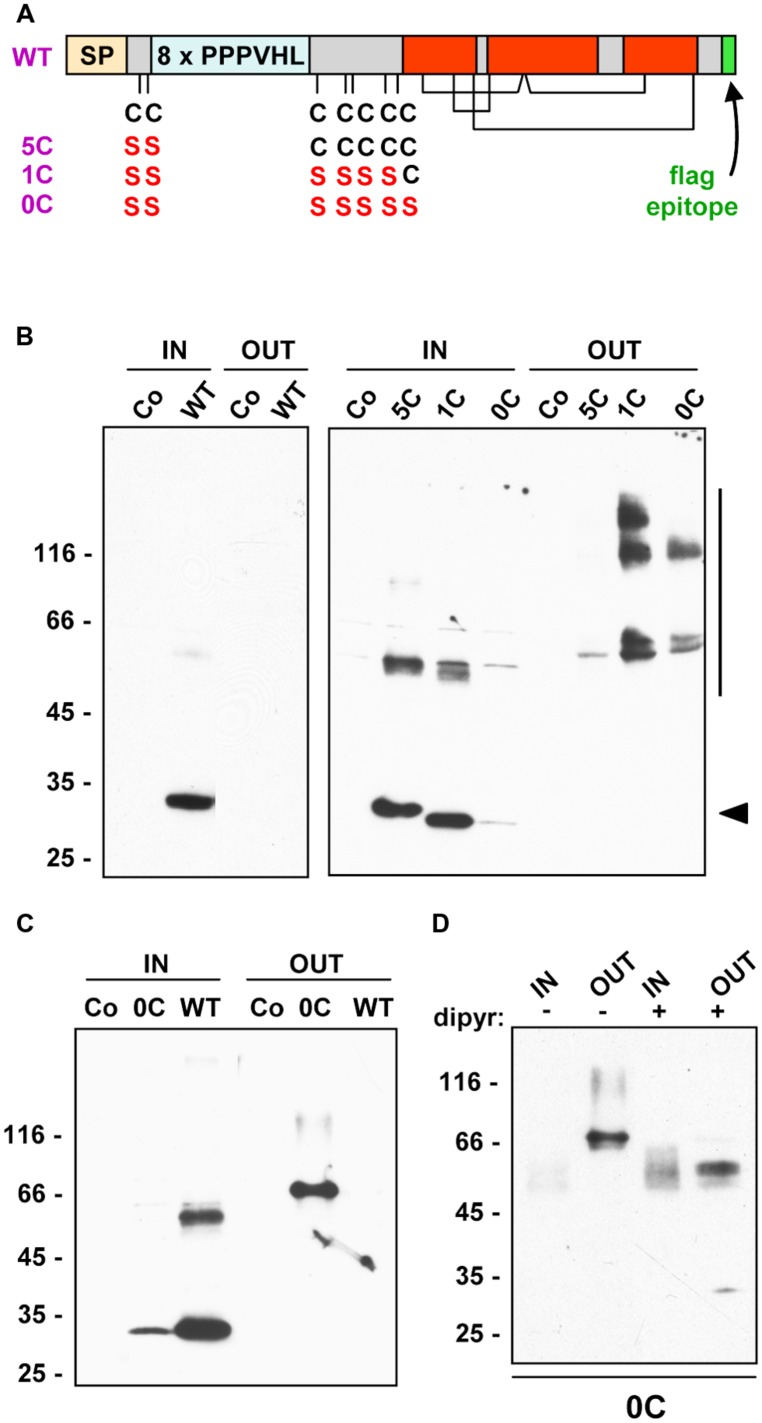
**Synthesis and secretion of γ-zein constructs. (A)** Schematic drawing of the different protein constructs, in which the following features are indicated: signal peptide (orange, SP); Cys residues in the N-terminal region and their substitutions with Ser in the mutated forms; repeated domain (light blue, 8 × PPPVHL); 2S albumin homologous domains (red), with the four putative intrachain disufide bonds; added flag epitope (green). **(B,C)** Tobacco protoplasts were transfected with plasmid containing the indicated γ-zein constructs or with empty plasmid as control (Co) and incubated for 24 h. Protoplasts (IN) or incubation media (OUT) were then homogenated in the presence of 2ME. Aliquots corresponding to equal number of protoplasts or the corresponding incubation medium were analyzed by SDS-PAGE in reducing conditions and protein blot with anti-flag antiserum. **(D)** Protoplasts transfected with plasmid encoding 0C-γ-zein were incubated for 24 h in the presence (+) or absence (-) of 0.25 mM 2,2′-dipyridyl (dipyr). Proteins were then analyzed as in **(B)**. In **(B)**, the positions of monomers (arrowhead) and polymers (verical bar) are indicated at right. In **(B–D)**, numbers at left indicate the positions of molecular mass markers, in kD.

The 27 kD γ-zein (hereon termed γ-zein, for simplicity) is among the most ancient maize prolamins (Xu and Messing, 2008, 2009); it is therefore a good model to study the early events that may have caused a shift of accumulation from the vacuole to the ER. After co-translational removal of the signal peptide for entry into the ER, mature γ-zein consists of two major regions, each corresponding to about half of the polypeptide (Prat et al., 1985). As schematically illustrated in **Figure 1A**, the N-terminal region is characterized by eight repeats of the hexapeptide PPPVHL (the repeated hexapeptide is also VHLPPP, because the last of the eight PPPVHL sequences is followed by PPP) and seven Cys residues involved in inter-chain bonds. A synthetic (VHLPPP)_8_ peptide forms *in vitro* an amphipathic structure that has affinity with lipids derived from plant ER (Kogan et al., 2004). The C-terminal region is homologous to 2S albumins (Shewry et al., 1995), which are characterized by three domains named A, B, and C, containing eight Cys that form four inter-chain disulfide bonds (**Figure 1A**). Consistently, when a fusion between thioredoxin and the C-terminal domain of γ-zein was expressed in *E. coli*, these eight Cys residues formed the intra-chain bonds typical of 2S albumins (Ems-McClung et al., 2002). The presence of a repeated sequence, as well as the 2S albumin domains, are common features of many prolamins (Shewry et al., 1995).

Early study established that γ-zein is soluble only in the presence of reducing agents (Vitale et al., 1982). The N-terminal region is retained in the ER if the 2S albumin-like region is deleted, whereas efficient secretion occurs when most of the N-terminal region is deleted (Geli et al., 1994). When a fragment (amino acids 24–112, from the translation start) that includes the eight Pro-rich repeats and the first six Cys residues was fused to the C-terminus of the vacuolar 7S storage globulin of common bean, phaseolin, the chimeric construct, termed zeolin, formed polymers with the main features of γ-zein: they are insoluble unless reduced and accumulate as homotypic PB in the ER (Mainieri et al., 2004). The Zera sequence, which consists of amino acids 1–112 of γ-zein, similarly promotes the formation of PB in the ER when fused to the N-terminus of a number of proteins (Torrent et al., 2009). Collectively, these experiments indicate that the N-terminal region of γ-zein forms inter-chain disulfide bonds and contains information sufficient for ER retention, independently of its position in fusion proteins.

When the six Cys residues of zeolin were mutated to Ser, efficient secretion occurred from transiently transfected protoplasts (Pompa and Vitale, 2006). Consistently, *in vivo* treatment with the reducing agent 2-mercaptoethanol caused secretion of zeolin from protoplasts of transgenic tobacco (Pompa and Vitale, 2006). Therefore, the inter-chain disulfide bonds are necessary for zeolin retention in the ER. This result was confirmed and extended by mutagenesis of the fluorescent chimera Zera-ECFP (Llop-Tous et al., 2010): when all the six Zera Cys residues were mutated or all eight repeats were deleted, PB formation was totally impaired, leading to efficient secretion, indicating cooperation between hydrophobic interactions and disulfide bonds in the formation of PB. Progressive mutagenesis of Cys residues or deletion of the Pro-rich repeats caused parallel gradual decrease in the size and abundance of Zera-ECFP PBs and increased secretion. The first two N-terminal Cys residues were identified as critical, since their mutagenesis was sufficient for full secretion, as visualized by fluorescence microscopy (Llop-Tous et al., 2010).

Using the full length γ-zein as a model, we have studied here the relationships between prolamin solubility, assembly, and intracellular traffic. We found a progressive change in subcellular location from the ER, to the vacuole, to secretion, that casts light on the structural features that may have allowed the evolutionary shift of storage protein localization from the vacuole to the ER, thus giving rise to a new subcellular compartment.

## MATERIALS AND METHODS

### PLASMID CONSTRUCTION

To prepare WT γ-zein for transient expression and detection with anti-flag antiserum, plasmid pBSKS.G1L (Bellucci et al., 1997), which contains the coding sequence of 27 kD γ-zein, was PCR amplified with the following oligos: 5′-TGTAGTCGACATGAGGGTGTTGCTCGTTGCCCT-3′ (termed forward1, the SalI restriction site is underlined) and 5′-ACATGCATGCCTATCATTACTTGTCGTCGTCGTCCTTGTA- GTCGTGGGGGACACCGCCGGCAGCA-3′, (termed reverse1, the SphI restriction site is underlined, the reverse complement of the codons encoding the flag epitope DYKDDDDK is double underlined). The sequence was restricted with SalI and SphI and inserted into the transient expression vector pDHA (Tabe et al., 1995). 1C γ-zein was constructed starting from pDHAzeolin(Cys^-^), see Pompa and Vitale (2006), first by amplifying its zein domain with the oligos 5′-TGTAGTCGAC*ATG* AGGGTGTTGCTCGTTGCCCTCGCTCTCCTGGCTCTCGCT- GCGAGCGCCACCTCCACGCATACAAGCGGCGGTAGCGGA- TCTCAGCCA-3′ (forward2, the SalI site is underlined, the translation start ATG is in Italic, the codons that substitute the first two Cys with Ser are double underlined) and 5′-AAAACTGCAG TTGCGACGGACTAGGATGAGG-3′ (reverse2, the PstI site is underlined). The amplified sequence was restricted with SalI and PstI and used to substitute the corresponding fragment of WT γ-zein inserted into pDHA. To construct 5C γ-zein, WT γ-zein inserted into pDHA was amplified with the oligos forward2 and reverse1, restricted with SalI and SphI and reinserted into pDHA. 0C γ-zein was constructed using a QuickChange protocol, starting from 1C γ-zein inserted into pDHA, using the oligos 5′-CAACAGGGAACCTCCGGCGTTGGCAGC-3′ (forward3, the codon that substitutes the last Cys of the N-terminal domain of γ-zein with Ser is double underlined) and its reverse complement (reverse3).

### TRANSIENT EXPRESSION IN PROTOPLASTS

Protoplasts were isolated from small (4–7 cm) leaves of tobacco (*Nicotiana tabacum*) SR1 plants grown in axenic conditions and subjected to polyethylene glycol-mediated transfection as described (Pedrazzini et al., 1997). Unless otherwise stated in the Results section, 40 μg of plasmid were used to transform 10^6^ protoplasts. Transformed protoplasts were resuspended in K3 medium [Gamborg’s B5 basal medium with minimal organics (Sigma), supplemented with 750 mg/L CaCl_2_ 2H_2_O, 250 mg/L NH_4_NO_3_, 136.2 g/L sucrose, 250 mg/L xylose, 1 mg/L 6-benzylaminopurine, and 1 mg/L 1-naphthalenacetic acid, pH 5.5], supplemented with 150 μg/ml bovine serum albumin as a competing substrate for extracellular proteases, and were incubated in the dark at 25°C. After the desired time, the incubation medium containing secreted proteins was carefully collected with a 2 ml syringe and protoplasts were washed and concentrated by the addition of three volumes of ice-cold W5 medium (154 mM NaCl, 5 mM KCl, 125 mM CaCl_2_ 2H_2_O, and 5 mM glucose) and centrifugation at 60 *g* for 10 min. The incubation medium and the protoplast pellet were frozen in liquid nitrogen and stored at -80°C, but freezing of protoplasts was avoided when subcellular fractionation was performed. When needed, inhibitors of protein trafficking brefeldin A (BFA: Roche; final concentration 10 μg/ml) or wortmannin (Sigma–Aldrich; final concentration 10 μM), were added to the protoplast incubation medium 1 h after transfection and maintained at the same concentration throughout the incubation. To inhibit prolyl hydroxylation, transfected protoplasts were incubated in the presence of 0.25 mM 2,2′-dipyridyl (dipyr), a chemical that inhibits *in vivo* activity of prolyl-4-hydroxylase (Moriguchi et al., 2011).

### PROTEIN ANALYSIS

**FIGURE 2:**
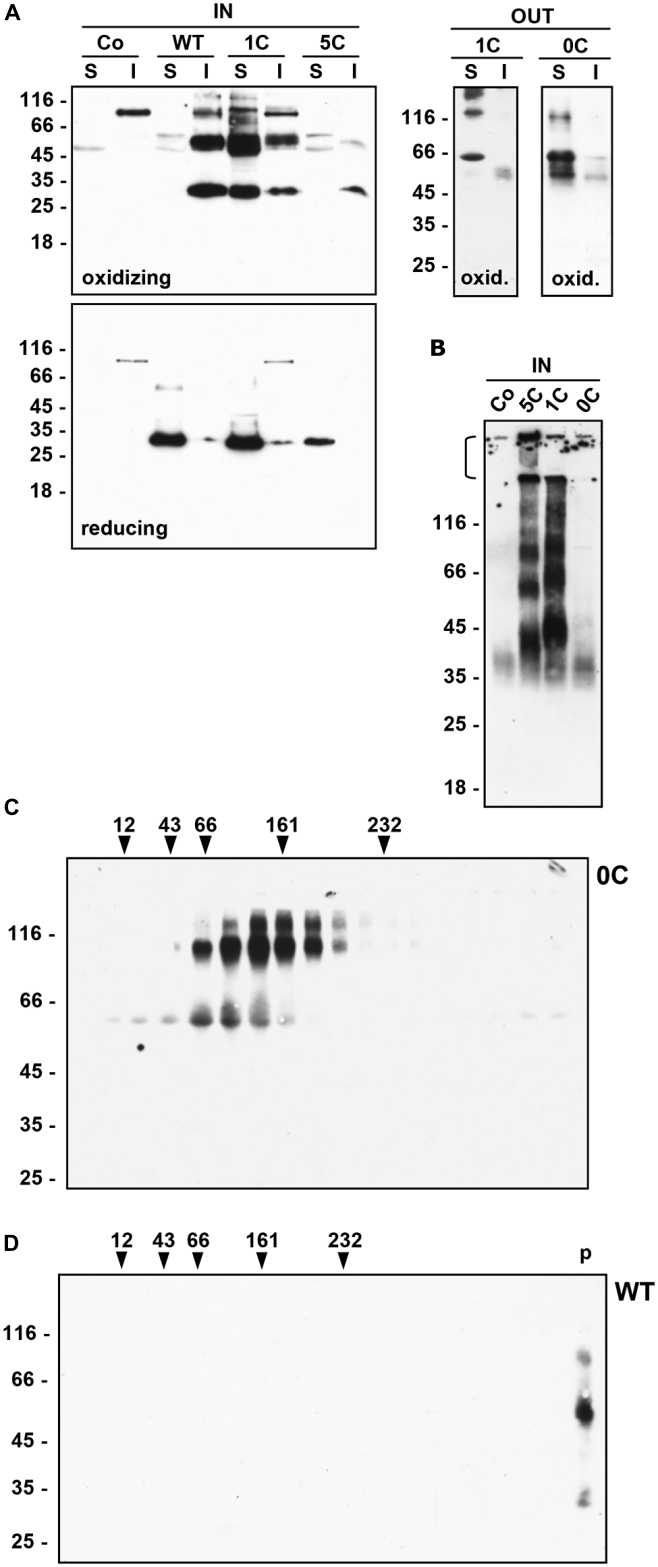
**Solubility and assembly of γ-zein constructs. (A)** Tobacco protoplasts were transfected with plasmid containing the indicated γ-zein constructs or with empty plasmid as control (Co) and incubated for 24 h. Protoplasts (IN) or incubation medium (OUT) were homogenated in the presence (reducing) or absence (oxidizing) of 2ME. After centrifugation, soluble (S) and insoluble (I) proteins were analyzed by SDS-PAGE in reducing conditions and protein blot with anti-flag antiserum. **(B)** Protoplasts transfected as in **(A)** were homogenated in the absence of 2ME. After centrifugation, soluble proteins were analyzed by SDS-PAGE in non-reducing conditions and protein blot with anti-flag antiserum. The stacking gel was not removed and is indicated by the bracket at left. **(C)** Protoplasts were transfected with plasmid encoding 0C-γ-zein. After 24 h the incubation medium was homogenated and loaded on top of a linear 5–25% (w/v) Suc gradient. After velocity separation of molecules by ultracentrifugation, an aliquot of each gradient fraction was analyzed by SDS-PAGE and protein blot with anti-flag antiserum. Top of the gradient is at left. Numbers on top indicate the position along the gradient and the molecular mass, in kD, of velocity centrifugation markers. **(D)** Protoplasts were transfected with plasmid encoding WT γ-zein. After 24 h protoplasts were homogenated and analyzed by velocity ultracentrifugation performed as in **(C)**. *p* indicates the material precipitated at the bottom of the tube. In all panels, numbers at left indicate the positions of molecular mass markers along the SDS-PAGE gel, in kD.

Protoplasts and incubation media were homogenated in homogenation buffer [150 mM Tris-Cl, pH 7.5, 150 mM NaCl, 1.5 mM EDTA, 1.5% Triton X-100, Complete protease inhibitor cocktail (Roche)], supplemented (reducing conditions) or not (oxidizing conditions) with 4% (v/v) 2-mercaptoethanol (2-ME). Separation of soluble and insoluble proteins was performed by centrifugation at 1,500 *g*, 10 min, 4°C. In the experiment shown in **Figure 2B**, the oxidizing homogenation buffer was supplemented with the alkylating agent iodoacetamide (Sigma–Aldrich, final concentration 70 mM). To analyze proteins, the homogenates were adjusted to 20 mM Tris-Cl pH 8.6, 1.0% SDS, 4% 2-ME, 8% glycerol (denaturation buffer), heated at 90°C for 5 min and separated by 15% acrylamide SDS-PAGE. For SDS-PAGE in non-reducing conditions, 2-ME was omitted from the denaturation buffer. Gels were blotted to Hybond-P membrane (GE Healthcare) and proteins revealed using with anti-flag rabbit polyclonal antibodies (Sigma–Aldrich; 1:2,000 dilution), anti-BiP rabbit antiserum (Pedrazzini et al., 1997, 1:10,000 dilution) or anti-endoplasmin rabbit antiserum (Klein et al., 2006, 1:2,500 dilution) and the Super-Signal West Pico Chemiluminescent Substrate (Pierce Chemical, Rockford, IL, USA). Protein Molecular Weight Markers (Fermentas, Vilnius, Lithuania) were used as molecular mass markers.

For velocity ultracentrifugation, the incubation medium of protoplasts expressing 0C γ-zein was homogenized with oxidizing homogenation buffer and centrifuged 1,500 *g*, 10 min, 4°C. The supernatant was loaded on top of a linear 5–25% (w/v) Suc gradient made in 150 mM NaCl, 1 mM EDTA, 0.1% Triton X-100, 50 mM Tris-Cl, pH 7.5. After centrifugation at 200,000 *g* average, 4°C for 20 h, an equal aliquot of each gradient fraction was analyzed by SDS-PAGE and protein blot. An identical gradient loaded with molecular mass markers was run in parallel.

### SUBCELLULAR FRACTIONATION AND VACUOLE PURIFICATION

Separation of microsomes derived from the different subcellular compartments was performed by protoplast homogenation in 10 mM KCl, 100 mM Tris-Cl, pH 7.8, 2 mM MgCl_2_, 12% (w/w) sucrose, followed by isopycnic centrifugation using linear 16–55% (w/w) sucrose gradient in the same buffer, as described (Mainieri et al., 2004). Isolation of vacuoles by floatation in Ficoll/betaine gradients (Dombrowski et al., 1994) and determination of the activity of the vacuolar marker α-mannosidase (Foresti et al., 2008) have also been described.

## RESULTS

### SOLUBILITY AND SECRETION OF γ-ZEIN ARE INVERSELY RELATED TO THE NUMBER OF Cys RESIDUES IN THE N-TERMINAL REGION AND THE EXTENT OF OLIGOMERIZATION

Three mutated constructs were produced, in which the first two, first six or all seven Cys residues of the N-terminal region were mutated to Ser (**Figure 1A**). The positions of these Cys residues, from the translation start, are 26, 28, 83, 101, 103, 111, and 117. To facilitate detection by protein blot, the flag epitope DYKDDDDK was added to the C-terminus of these constructs and of WT γ-zein. For brevity, here on the constructs will be called based on the number of remaining Cys residues of the N-terminal region: 5C indicates γ-zeinC^26,28^S, 1C indicates γ-zeinC^26,28,83,101,103,111^S, and 0C indicates γ-zeinC^26,28,83,101,103,111,117^S; WT indicates wild type γ-zein. All constructs were transiently expressed in tobacco leaf protoplasts under the control of the constitutive CaMV 35S promoter.

To first test whether the mutations promoted traffic of γ-zein, at 24 h after transfection proteins were extracted from protoplasts or the incubation media in the presence of non-ionic detergent and the reducing agent 2-mercaptoethanol (2-ME), separated by SDS-PAGE and detected with anti-flag antiserum. Polypeptides of the expected size, migrating between the 25 and 35 kD molecular mass markers (**Figure 1B**, arrowhead), as well as higher molecular mass forms that indicated oligomer formation or post-translational modifications, in part related to secretion (**Figure 1B**, vertical bar), were detected in protoplasts transfected with all γ-zein constructs, but not when transfection was performed with the empty plasmid. WT γ-zein was exclusively recovered in protoplasts, as expected, whereas a progressive decrease in the number of Cys residues caused a parallel, gradual increase in secretion (**Figure 1B**). In the experiment shown in **Figure 1B**, secretion of 0C-γ-zein was almost complete. This was not always the case in fully independent transfections, probably reflecting variability in intracellular redox balance among the different protoplast preparations (Lombardi et al., 2012), but the ratio of secreted/intracellular recombinant protein was always 0C > 1C > 5C ≥ WT γ-zein, where secreted WT γ-zein was always virtually 0% (**Table 1**). Even in experiments in which the total amount of WT γ-zein synthesized was clearly higher than that of 0C, the former was not secreted at all, indicating that the differences in the traffic properties are not dependent on expression levels (**Figure 1C**).

**Table 1:** Secretion levels of γ-zein constructs.

γ-zein construct	WT	5C	1C	0C
	**Percentage of recombinant protein that was secreted**			
Experiment 1^a^	0	0	5	79
Experiment 2	0	10	68	90
Experiment 3	0	0	32	51

*^a^Experiments 1, 2, and 3 are three fully independent protoplast preparation and transfection experiments.*

The γ-zein forms migrating around 50–60 kD could represent dimers particularly difficult to denature, whereas the larger forms, that are mainly detected extracellularly, could indicate further oligomerization or post-translational modifications related to traffic (**Figure 1B**). Higher molecular mass forms difficult to denature have also been detected to variable extents upon expression of γ-zein or protein fusions containing the N-terminal γ-zein domain in transgenic plants (Geli et al., 1994; Bellucci et al., 2000; Mainieri et al., 2004; Torrent et al., 2009; Virgili-López et al., 2013).

Some of the γ-zein oligomerizations/modifications are clearly dependent on the presence of Cys residues in the N-terminal domain, as can be appreciated comparing secreted 1C- and 0C-γ-zein, but others are not (**Figure 1B**). It has been suggested that hydroxylation of proline residues could occur on γ-zein (Geli et al., 1994). *In vivo* inhibition of prolyl-4-hydroxylase abolished most of the differences between intracellular and secreted 0C-γ-zein (**Figure 1D**), indicating that the increase in molecular mass beyond 50–60 kD was indeed dependent on this modification, possibly followed by O-glycosylation of hydroxyproline occurring only along the route to secretion or extracellularly.

The inverse relationship between the number of Cys residues and secretion suggested that the removal of Cys residues increases γ-zein solubility in the naturally oxidizing ER environment. Homogenation in the absence or presence of 2-ME was therefore performed (**Figure 2A**, oxidizing and reducing, respectively), followed by centrifugation to separate soluble and insoluble material (S and I samples in **Figure 2A**), denaturation of all samples and analysis by SDS-PAGE in reducing conditions. Putative dimers were much more evident if extraction was performed in oxidizing conditions. WT γ-zein was insoluble unless extracted in the presence of reducing agent, as expected (Vitale et al., 1982), whereas decreasing the number of Cys residues caused a parallel increase in solubility: a very small proportion of intracellular 5C, more than 50% of intracellular 1C, and more than 90% of secreted 0C- and 1C-γ-zein were solubilized also in the absence of reducing agent (**Figure 2A**, compare S and I for each sample).

Analysis by non-reducing SDS-PAGE indicated that soluble 1C-γ-zein and, even more, 5C-γ-zein undergo oligomerization beyond the dimer stage (**Figure 2B**; notice that a relevant proportion of 5C γ-zein does not even enter the separating gel). Oligomerization of the very small proportion of intracellular 0C-γ-zein was difficult to determine (notice that the diffused band around 40 kD is present also in the control and is thus irrelevant), but the native molecular mass of secreted 0C γ-zein polypeptides analyzed by velocity gradient ultracentrifugation corresponded roughly to that determined by SDS-PAGE, indicating that dimers particularly difficult to denature are formed by all constructs and that 0C-γ-zein does not form larger oligomers beyond dimers (**Figure 2C**). WT γ-zein could not be analyzed by non-reducing SDS-PAGE because of its insolubility, but upon velocity centrifugation it migrated at the bottom of the tube, indicating that it formed very large disulfide-bonded polymers, as expected (**Figure 2D**).

It can be concluded that γ-zein expressed in leaf protoplasts is insoluble unless reduced, as in maize endosperm, and that lowering the number of Cys residues progressively reduces the ability to form large disulfide-bonded polymers, resulting in increased solubility and ability to traffic along the secretory pathway.

### SOLUBLE γ-ZEIN TRAFFICS IN PART TO THE VACUOLE UNLESS ALL N-TERMINAL Cys RESIDUES ARE MUTATED

**FIGURE 3:**
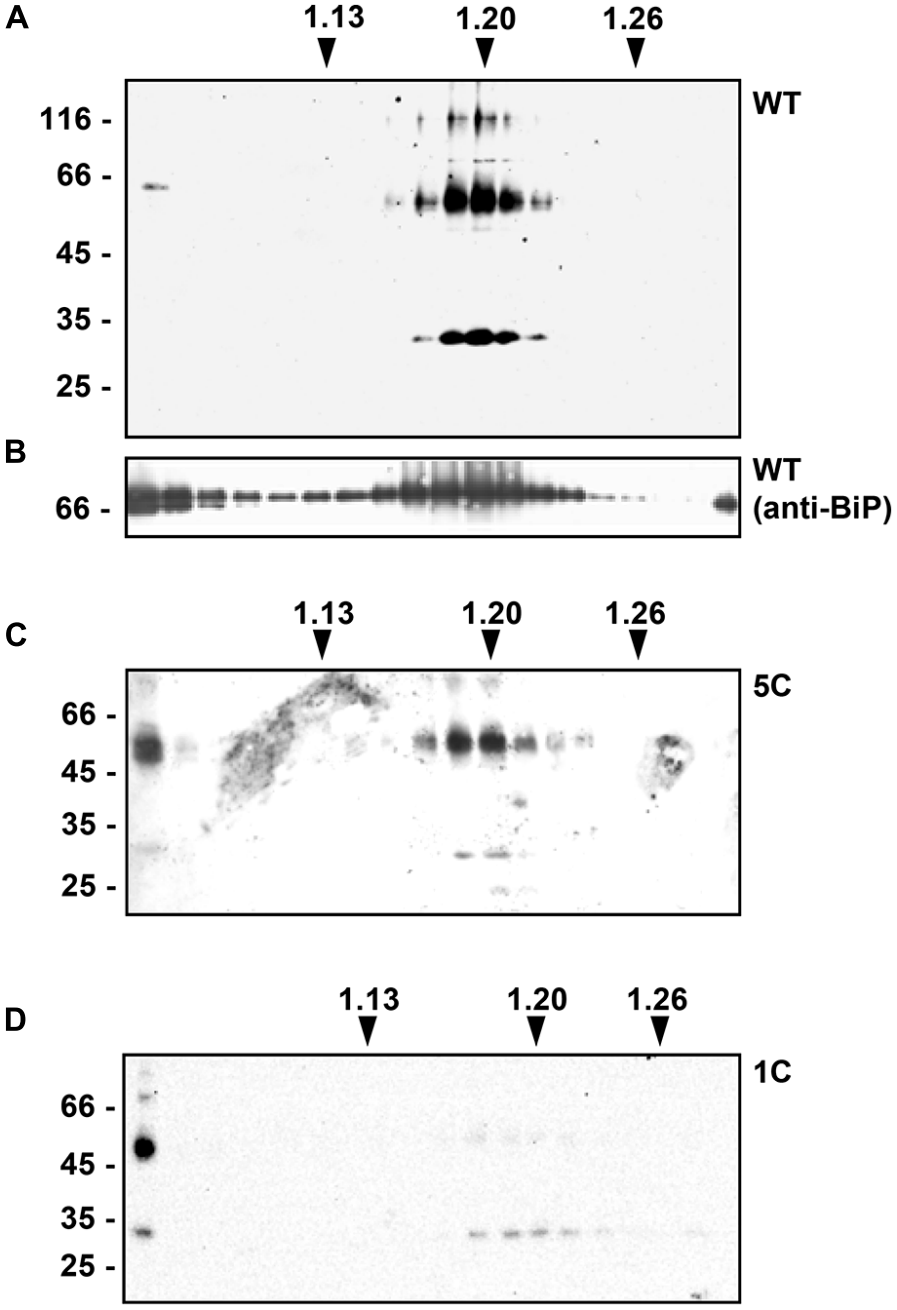
**Subcellular distribution of the different γ-zein constructs.** Tobacco protoplasts were transfected with plasmid encoding WT γ-zein **(A,B)**, 5C-γ-zein **(C)** or 1C-γ-zein **(D)**. After 24 h incubation, protoplasts were homogenated in oxidizing conditions, in the absence of detergent and presence of sucrose. Homogenates were fractionated by isopycnic ultracentrifugation on sucrose density gradients. Equal amounts of each gradient fraction were analyzed by SDS-PAGE and protein blot using anti-flag **(A,C,D)** or anti-BiP **(B)** antiserum. Numbers on top indicate fraction density (g/ml). In each panel, numbers at left indicate the positions of molecular mass markers, in kD.

**FIGURE 4:**
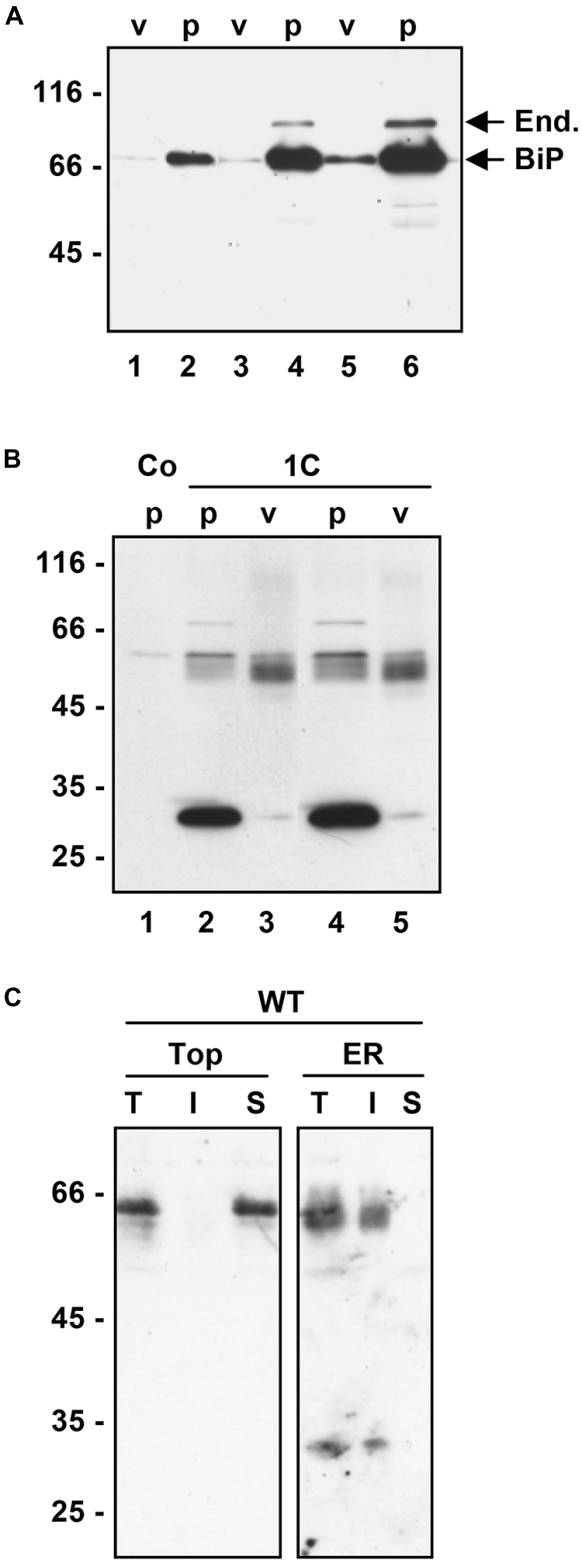
**Vacuole purification. (A)** Tobacco protoplasts were transfected with empty plasmid. After 24 h incubation, either protoplast homogenates (p) or isolated vacuoles (v) were prepared and analyzed by SDS-PAGE and protein blot using a mixture of anti-BiP and anti-endoplasmin antisera. Lane 1 and 2: equal number of protoplasts and vacuoles, lanes 3 and 4 equal activity of the vacuolar enzyme α-mannosidase, lanes 5 and 6 fivefold amount of material with respect to lanes 3 and 4, respectively. **(B)** Tobacco protoplasts were transfected with empty plasmid (Co) or plasmid encoding 1C-γ-zein. After 24 h incubation, either protoplast homogenates (p) or isolated vacuoles (v) were prepared and analyzed by SDS-PAGE and protein blot using anti-flag antiserum. Lane 1-3: equal number of protoplasts and vacuoles, lanes 4 and 5 equal activity of the vacuolar enzyme α-mannosidase. **(C)** Tobacco protoplasts were transfected with plasmid encoding WT γ-zein. After 24 h incubation, protoplasts were homogenated in oxidizing conditions, in the absence of detergent and presence of sucrose. The homogenate was fractionated by isopycnic ultracentrifugation on sucrose density gradient. The fractions remaining on top of the gradient (top) or containing ER microsomes (ER) were diluted with homogenation buffer in oxidizing conditions and centrifuged. Equal amounts of total homogenate (T), soluble material (S) or insoluble precipitate (I) were analyzed by SDS-PAGE and protein blot using anti-flag antiserum. In each panel, numbers at left indicate the positions of molecular mass markers, in kD.

To determine whether the introduced mutations also changed the intracellular distribution of γ-zein polypeptides that were not secreted during the 24 h incubation, transfected protoplasts were homogenized in the absence of detergent and the homogenate subjected to isopycnic gradient centrifugation to separate subcellular compartments. Protein blots were analyzed using either anti-flag serum or serum against the chaperone BiP, a major soluble protein of the ER. WT γ-zein migrated as a clear peak in the region around density 1.20 g/L, which contained also BiP molecules (**Figures 3A,B**). This is an expected behavior for a protein forming PBs in the ER. An extremely low proportion of putative dimers migrated at top of the gradient (**Figure 3A**, first lane at left, faint band below the 66 kD marker). The chaperone BiP is commonly also found at top of isopycnic gradients, possibly due to partial *in vitro* release from broken ER membranes before they seal into microsomes (**Figure 3B**; see Gomord et al., 1997; Frigerio et al., 2001). Both 5C-γ-zein (**Figure 3C**) and 1C-γ-zein (**Figure 3D**) formed a peak at the same density of WT γ-zein, but they were also present at top of the gradients in much more relevant proportions compared to WT γ-zein, more markedly in the case of 1C- than 5C-γ-zein. This could indicate *in vitro* release from the ER; however, leaf vacuoles completely break during homogenation and release their soluble content, which remains on top of isopycnic gradients together with cytosolic soluble proteins (Pedrazzini et al., 1997; Frigerio et al., 2001). We therefore verified whether mutated, soluble γ-zein polypeptides are in part delivered to the vacuole. Protoplasts were subjected to gentle lysis, followed by centrifugation in buffers that allow vacuole floatation. These preparations of intact vacuoles contain very low proportions of the major soluble ER chaperones BiP and endoplasmin, indicating almost no contamination by microsomes originating from other endomembranes (**Figure 4A**). Vacuoles prepared from protoplasts expressing 1C-γ-zein were highly enriched in γ-zein dimers, indicating that this mutated protein is in part delivered to the vacuole (**Figure 4B**) and suggesting that also 5C- and WT γ-zein dimers present at top of isopycnic gradients (**Figure 3**) are located in the vacuole. Consistently, the extremely low amount of WT γ-zein dimers at top of isopycnic gradients (see the first lane in **Figure 3A**) are fully soluble in the absence of reducing agent, whereas those in the ER fraction are insoluble (**Figure 4C**). This indicates that WT γ-zein polypeptides can, albeit extremely rarely, remain soluble and available for vacuolar delivery.

### SECRETION OF SOLUBLE γ-ZEIN IS INHIBITED BY BREFELDIN A AND STIMULATED BY WORTMANNIN

**FIGURE 5:**
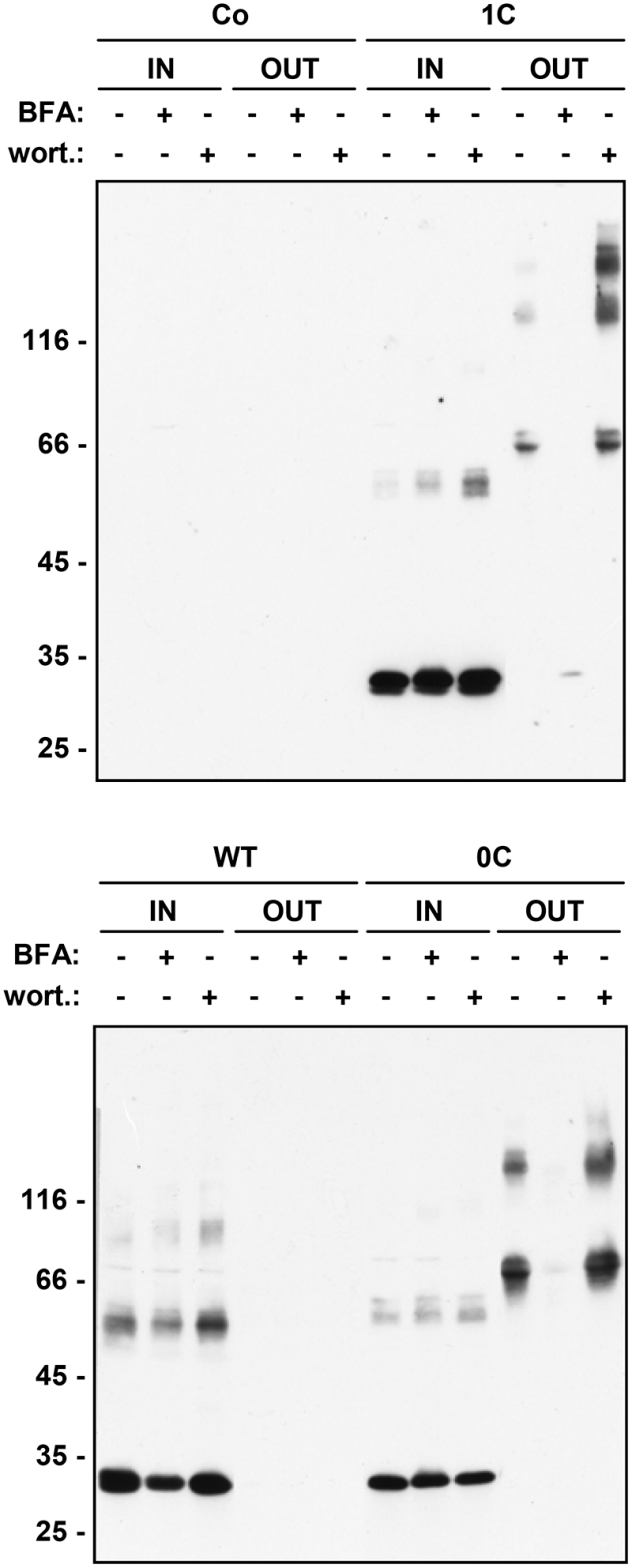
**Effect of intracellular traffic inhibitors on the secretion of γ-zein constructs.** Tobacco protoplasts were transfected with plasmid containing the indicated γ-zein construct or with empty plasmid as control (Co). After 24 h incubation in the presence (+) or absence (-) of brefeldin A (BFA) or wortmannin (wort.), aliquots corresponding to equal number of protoplasts (IN) or the corresponding incubation medium (OUT) were analyzed by SDS-PAGE in reducing conditions, followed by protein blot using anti-flag antiserum. In each panel, numbers at left indicate the positions of molecular mass markers, in kD.

Most soluble seed storage proteins are delivered to storage vacuoles following a traffic pathway that involves the Golgi apparatus and multivesicular bodies (MVB, also termed prevacuolar compartment). The chemicals BFA and wortmannin affect this pathway: BFA blocks the traffic step from the ER to the Golgi apparatus and therefore inhibits vacuolar delivery as well as Golgi-mediated protein secretion (Gomez and Chrispeels, 1993; Jones and Herman, 1993; Pedrazzini et al., 1997); wortmannin inhibits the recycling of vacuolar sorting receptors from MVB to the Golgi/*trans* Golgi network, thus inducing default secretion of soluble proteins that use these receptors to reach vacuoles (daSilva et al., 2005). However, highly condensed insoluble prolamins can be delivered to vacuoles by autophagy (Levanony et al., 1992; Coleman et al., 1996; Reyes et al., 2011); furthermore, vacuoles can also be reached by BFA-insensitive vesicular traffic pathways that seem to bypass the Golgi apparatus or MVB (Gomez and Chrispeels, 1993; Hara-Nishimura et al., 1998; Pedrazzini et al., 2013). We have therefore investigated the effects of BFA and wortmannin on γ-zein trafficking. Treatment of protoplasts with BFA fully inhibited the secretion of 1C- and 0C-γ-zein as well as the modifications that increase the molecular mass of dimers, indicating that secretion occurs via the Golgi apparatus and that the post-translational modifications require traffic (**Figure 5**). There was no evident increase in accumulation of intracellular polypeptides, most probably because the drug also partially inhibits protein synthesis (Mellor et al., 1994; de Virgilio et al., 2008); consistently, WT γ-zein, which is not secreted by untreated protoplasts, accumulated at lower amounts upon BFA treatment (**Figure 5**). Wortmannin markedly stimulated the secretion of 1C- and to a much less extent that of 0C-γ-zein, whereas no WT γ-zein could be detected in the protoplast incubation medium even upon treatment with this drug (**Figure 5**). This indicates that the vacuolar delivery of a relevant proportion of 1C-γ-zein polypeptides occurs through the same pathway followed by most vacuolar storage proteins. In agreement with the other data presented in this study, the results of wortmannin treatment also indicate that WT γ-zein does not enter this traffic pathway and that traffic of 0C-γ-zein leads mainly to secretion rather than vacuolar delivery.

## DISCUSSION

In all cereals, PBs contain the products of many different prolamin genes. Detailed characterization of maize seed development showed that zein genes have distinct temporal patterns of expression (Woo et al., 2001) and produce proteins with distinct ordered locations within individual PBs (Lending and Larkins, 1989). Characterization of the *floury1* (Holding et al., 2007) and *opaque1* (Wang et al., 2012) mutations indicated that specific proteins of the ER membrane and of the acto-myosin system are also involved in the shaping of PBs with normal size and morphology. These features indicate that a natural PB is assembled through a sequence of complex molecular interactions, whose details remain in large part unknown. The discovery that the ectopic expression of certain individual prolamins is sufficient to form large electron-dense accretions in the ER has, however, opened the way to a reductionist approach toward understanding prolamin assembly into polymers and their ER retention (Geli et al., 1994; Bagga et al., 1995). The 27 kD γ-zein, perhaps the best characterized of these proteins, is among the most ancient prolamins (Xu and Messing, 2008, 2009), is synthesized starting at very early stages of seed development and is specifically localized at the PB periphery, in proximity to the ER membrane, in mature PBs (Lending and Larkins, 1989). Elimination of 27 kD γ-zein by RNAi or induced gene deletion does not abolish maize PB formation, but strongly alters their morphology, size, and number, and has a general effect on endosperm texture (Wu and Messing, 2010; Yuan et al., 2014). Therefore, 27 kD γ-zein is both sufficient to form a homotypic PB and necessary for the normal assembly of a natural PB. Altogether, the features described above make 27 kD γ-zein an excellent model to define the structural requirements to initiate PB formation.

### DISULFIDE BONDS, INSOLUBILITY AND INABILITY TO TRAFFIC

Deletions of γ-zein domains (Geli et al., 1994), amino acid substitutions carried out on fusions between the γ-zein N-terminal domain and otherwise soluble proteins (Pompa and Vitale, 2006; Llop-Tous et al., 2010), as well as *in vivo* treatments with reducing agent (Pompa and Vitale, 2006), all pointed to the importance of the Cys residues in the N-terminal region of the polypeptide in promoting γ-zein insolubility and ER retention, but this had not been demonstrated for the full-length protein. The results reported here demonstrate that 0C-γ-zein is fully soluble and very efficiently secreted, indicating that the presence of the C-terminal, 2S albumin-like domain and the N-terminal repeated region are not sufficient to form insoluble polymers and for ER retention in the absence of the seven N-terminal Cys residues. This efficient secretion also indicates that 0C-γ-zein is not a misfolded protein disposed by ER quality control. Indeed all constructs studied here are able to form dimers, suggesting a similar folding pathway. Forms that could represent dimers have also detected in transgenic plants expressing γ-zein (Geli et al., 1994; Bellucci et al., 2000) as well as upon expression of zeolin (Mainieri et al., 2004) and Zera (Torrent et al., 2009) fusions. Intracellular dimers and further polymers are detected in much higher proportions when extraction is performed in the absence of reducing agent. The detection of dimers of 0C-γ-zein and polymers of 1C-γ-zein suggest that either the intra-chain disulfide bonds of the 2S albumin-like region allow an overall conformation that favors inter-chain hydrophobic interactions or, perhaps surprisingly, the Cys residues present in this region are also directly involved in inter-chain bonds.

The results presented here confirm those obtained by Zera-ECFP mutagenesis regarding the direct relationship between number of Cys residues in the N-terminal domain and ability to form ER-located PB (Llop-Tous et al., 2010). However, the high importance of the first two N-terminal Cys in Zera-ECFP was not confirmed in full-length γ-zein: mutagenesis of these residues in Zera-ECFP resulted in full secretion (Llop-Tous et al., 2010), whereas 5C γ-zein is largely insoluble and ER-located, and it is almost unavailable for secretion. This indicates that the contribution of individual Cys residues of the N-terminal region to PB formation are less strict in full length γ-zein, as also suggested by domain deletion experiments (Geli et al., 1994).

As mentioned in the Introduction, synthetic (VHLPPP)_8_ forms an amphipathic helix that interacts *in vitro* both with itself and with liposomes (Kogan et al., 2004). The inter-chain disulfide bonds may thus determine folding properties that stabilize and further promote similar interactions *in vivo*. The affinity with lipids may explain the natural position of γ-zein at the periphery of the PB, in close contact with the luminal face of the ER membrane, its ability form stable PBs also when expressed alone, and the altered PB shape in maize mutants that lack γ-zein. Thus, even if the very abundant α-zeins also assemble into disulfide bonded polymers and do not seem to interact covalently with γ-zein (Vitale et al., 1982), the latter seems fundamental to provide a scaffold that favors the stable architecture of a natural PB.

### POLYMERIZATION AND VACUOLAR SORTING

As two, six or all seven Cys residues of the N-terminal domain were mutated, a parallel increase in BFA-sensitive secretion was observed. However, unlike WT γ-zein, intracellular 5C- and 1C-γ-zein were not exclusively located in the ER. The presence of a relevant proportion of protein at top of isopycnic gradients suggested a vacuolar localization, confirmed by vacuole isolation. The polypeptides delivered to the vacuole are soluble. Treatments with wortmannin indicated that this vacuolar delivery is due to traffic rather than autophagy and that the route is the same followed by most 2S albumins and 7/11S globulins. Very efficient secretion of 0C- and partial vacuolar sorting of 1C-γ-zein indicate that Cys^117^ acts as a determinant for vacuolar sorting. These results support a model of the evolution of PB formation from the mechanism of storage protein sorting to vacuoles and not directly from default secretion.

The γ-zein fragments used in zeolin and Zera constructs never included Cys^117^, because they stopped a few amino acids ahead of it. We do not know whether any other Cys residue of the N-terminal region would be sufficient in promoting vacuolar sorting of full-length γ-zein, but the following observations indicate that this promotion occurs because the Cys residue stabilizes polymerization events that require other γ-zein domains. First, a γ-zein deletion construct that lacks most of the N-terminal region is very efficiently secreted but still contains Cys^26^, Cys^28^, and Cys^117^ (Geli et al., 1994), indicating that these residues are not sufficient *per se* to drive vacuolar sorting or ER retention. Second, experiments performed on the bean 7S globulin phaseolin indicate that the short C-terminal hydrophobic peptide that acts as a vacuolar sorting signal and promotes transient polymerization events (Frigerio et al., 1998; Holkeri and Vitale, 2001; Castelli and Vitale, 2005) can be at least in part replaced by a Cys residue that forms an inter-chain bond (Pompa et al., 2010). There is thus a striking similarity between the effect of the presence of a single additional Cys in 1C- compared to 0C-γ-zein and the artificial addition of a single Cys to a mutated, secreted phaseolin: in both cases vacuolar sorting is stimulated. It should be underlined that addition of Cys residues in phaseolin domains distant from the C-terminal end does not have any effect, indicating that disulfide bonds are formed when close interactions anyway exist, as expected (Pompa et al., 2010). In γ-zein, these interactions clearly require the Pro-rich amphipathic, repeated region.

### AN EVOLUTIONARY MODEL

**FIGURE 6:**
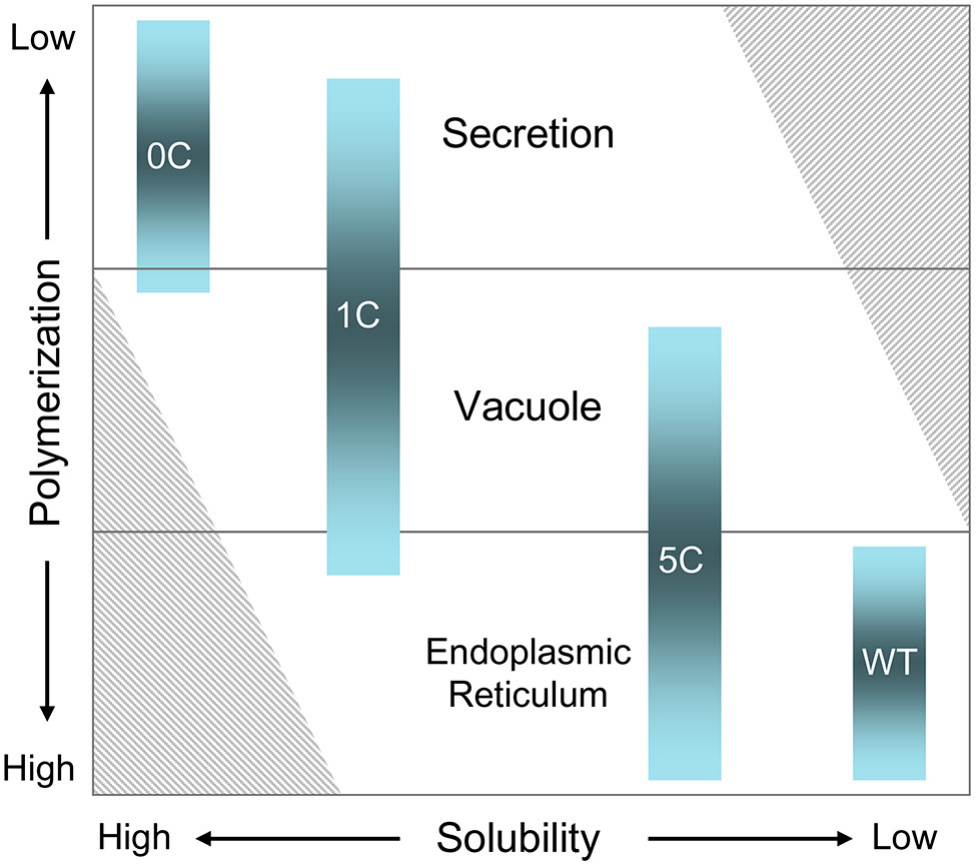
**A model for the relationships between solubility, polymerization and subcellular localization of seed storage proteins, based on the behaviors of WT, 5C-, 1C-, and 0C-γ-zein.** The shaded columns indicate the subcellular localization of each construct, where the relative space occupied in each of the three locations is roughly proportional with the observed average distributions. The striped areas at lower left and upper right indicate situations predicted to be impossible. For simplicity, the model does not take into consideration other factors that determine or influence the subcellular localization, such as for example the short vacuolar sorting signals identified in several soluble 2S albumins and 7/11S globulins.

Prolamins have been divided into three groups, named I, II, and III starting from the most recently evolved (Xu and Messing, 2009). The γ- and β-zeins have been assigned to group II. Their synthesis during seed development starts before that of the α- and δ-zeins, which belong to group I (Lending and Larkins, 1989). Maize does not have group III members. The 27 kD γ-zein is therefore among the most ancient maize prolamins (Xu and Messing, 2008, 2009). The A, B, and C domains that are common to 2S albumins and to other vacuolar seed proteins – such as trypsin inhibitor – are the most common feature of group II and III prolamins, indicating an evolution involving regions of vacuolar proteins (Xu and Messing, 2008). It has been hypothesized that conversion of Cys residues of the A, B, or C domains from an involvement in intra-chain disulfide bonds to inter-chain bonds may have been important events in this evolution (Kawagoe et al., 2005). The results shown here indicate a different model, in which at least one of the key mechanisms was the addition of new Cys residues that further increase and stabilize inter-chain interactions otherwise involved in vacuolar sorting (**Figure 5**). According to this model, the progressive addition reached a level that promoted the formation of large, insoluble polymers unable to traffic out of the ER (**Figure 6**).

The ER is the port of entry of the secretory pathway; intuitively, the development of a mechanism for protein accumulation in this compartment would be both more rudimentary and more energy-saving than using protein trafficking and sorting to vacuoles. However, the very complex array of players and molecular interactions involved in productive protein folding and quality control in the ER, the main functions of this compartment, may be negatively affected, at least in theory, by the presence of large amounts of stored material. Evolution of proteins that accumulate in the ER as insoluble polymers, apparently a very unusual event in any kingdom, may have been favored by the fact that the endosperm, unlike cotyledons, undergoes programmed cell death at the late stages of cereal seed development, making thus irrelevant the final location of stored proteins for what regards their mobilization during germination. It should, however, be underlined that the many experiments in which proteins that accumulate in the ER were ectopically expressed in vegetative tissues indicate that the plant ER is anyway very tolerant for what regards protein accumulation (Herman, 2008), another feature that may have allowed protein body formation in plants but not in members of other kingdoms.

## Conflict of Interest Statement

The authors declare that the research was conducted in the absence of any commercial or financial relationships that could be construed as a potential conflict of interest.

## References

[B1] Adachi M. Takenaka Y. Gidamis A. B. Mikami B. Utsumi S. (2001). Crystal structure of soybean proglycinin alaB1b homotrimer. *J. Mol. Biol.* 305 291–305 10.1006/jmbi.2000.4310 11124907

[B2] Anelli T. Sitia R. (2010). Physiology and pathology of proteostasis in the early secretory compartment. *Semin. Cell Dev. Biol.* 21 520–525 10.1016/j.semcdb.2010.02.006 20178856

[B3] Bagga S. Adams H. Kemp J. D. Sengupta-Gopalan C. (1995). Accumulation of 15-kilodalton zein in novel protein bodies in transgenic tobacco. *Plant Physiol.* 107 13–23 12228338 10.1104/pp.107.1.13 PMC161159

[B4] Bellucci M. Alpini M. Arcioni S. (2000). Expression of maize γ-zein and β-zein genes in transgenic *Nicotiana tabacum* and *Lotus corniculatus*. *Plant Cell Tissue Organ Cult.* 62 141–151 10.1023/A:1026735106843

[B5] Bellucci M. Lazzari B. Viotti A. Arcioni S. (1997). Differential expression of a γ-zein gene in *Medicago sativa*, *Lotus corniculatus and Nicotiana tabacum*. *Plant Sci.* 127 161–169 10.1016/S0168-9452(97)00092-7

[B6] Castelli S. Vitale A. (2005). The phaseolin vacuolar sorting signal promotes transient, strong membrane association and aggregation of the bean storage protein in transgenic tobacco. *J. Exp. Bot.* 56 1379–1387 10.1093/jxb/eri139 15809284

[B7] Coleman C. E. Herman E. M. Takasaki K. Larkins B. A. (1996). The maize gamma-zein sequesters alpha-zein and stabilizes its accumulation in protein bodies of transgenic tobacco endosperm. *Plant Cell* 8 2335–2345 10.1105/tpc.8.12.2335 8989886 PMC161356

[B8] daSilva L. L. Taylor J. P. Hadlington J. L. Hanton S. L. Snowden C. J. Fox S. J. (2005). Receptor salvage from the prevacuolar compartment is essential for efficient vacuolar protein targeting. *Plant Cell* 17 132–148 10.1105/tpc.104.026351 15632053 PMC544495

[B9] de Virgilio M. De Marchis F. Bellucci M. Mainieri D. Rossi M. Benvenuto E. (2008). The human immunodeficiency virus antigen Nef forms protein bodies in leaves of transgenic tobacco when fused to zeolin. *J. Exp. Bot.* 59 2815–2829 10.1093/jxb/ern143 18540021 PMC2486477

[B10] Dombrowski J. E. Gomez L. Chrispeels M. J. Raikhel N. V. (1994). “Targeting of proteins to the vacuole,” in *Plant Molecular Biology Manual* eds Gelvin S. B. Schilperoort R. A. (Dordrecht: Kluwer Academic Publishers) 1–29

[B11] Ems-McClung S. C. Benmoussa M. Hainline B. E. (2002). Mutational analysis of the maize gamma zein C-terminal cysteine residues. *Plant Sci.* 162 131–141 10.1016/S0168-9452(01)00549-0

[B12] Foresti O. De Marchis F. de Virgilio M. Klein E. M. Arcioni S. Bellucci M. (2008). Protein domains involved in assembly in the endoplasmic reticulum promote vacuolar delivery when fused to secretory GFP, indicating a protein quality control pathway for degradation in the plant vacuole. *Mol. Plant* 1 1067–1076 10.1093/mp/ssn066 19825604

[B13] Frigerio L. de Virgilio M. Prada A. Faoro F. Vitale A. (1998). Sorting of phaseolin to the vacuole is saturable and requires a short C-terminal peptide. *Plant Cell* 10 1031–1042 10.1105/tpc.10.6.1031 9634590 PMC144029

[B14] Frigerio L. Pastres A. Prada A. Vitale A. (2001). Influence of KDEL on the fate of Trimeric or assembly-defective phaseolin: selective use of an alternative route to vacuoles. *Plant Cell* 13 1109–1126 10.1105/tpc.13.5.1109 11340185 PMC135559

[B15] Geli M. I. Torrent M. Ludevid D. (1994). Two structural domains mediate two sequential events in γ-zein targeting: protein endoplasmic reticulum retention and protein body formation. *Plant Cell* 6 1911–1922 12244234 10.1105/tpc.6.12.1911 PMC160571

[B16] Gomez L. Chrispeels M. J. (1993). Tonoplast and soluble vacuolar proteins are targeted by different mechanisms. *Plant Cell* 5 1113–1124 10.1105/tpc.5.9.1113 12271099 PMC160345

[B17] Gomord V. Denmat L. A. Fitchette-Lainé A. C. Satiat Jeunemaitre B. Hawes C. Faye L. (1997). The C-terminal HDEL sequence is sufficient for retention of secretory proteins in the endoplasmic reticulum (ER) but promotes vacuolar targeting of proteins that escape the ER. *Plant J.* 11 313–325 10.1046/j.1365-313X.1997.11020313.x 9076996

[B18] Gu Y. Q. Wanjugi H. Coleman-Derr D. Kong X. Anderson O. D. (2010). Conserved globulin gene across eight grass genomes identify fundamental units of the loci encoding seed storage proteins. *Funct. Integr. Genomics* 10 111–122 10.1007/s10142-009-0135-x 19707805

[B19] Hara-Nishimura I. Shimada T. Hatano K. Takeuchi Y. Nishimura M. (1998). Transport of storage proteins to protein storage vacuoles is mediated by large precursor-accumulating vesicles. *Plant Cell* 10 825–836 10.1105/tpc.10.5.825 9596640 PMC144021

[B20] Herman E. M. (2008). Endoplasmic reticulum bodies: solving the insoluble. *Curr. Opin. Plant Biol.* 11 672–679 10.1016/j.pbi.2008.08.004 18824401

[B21] Holding D. R. Otegui M. S. Li B. Meeley R. B. Dam T. Hunter B. G. (2007). The maize floury1 gene encodes a novel endoplasmic reticulum protein involved in zein protein body formation. *Plant Cell* 19 2569–2582 10.1105/tpc.107.053538 17693529 PMC2002605

[B22] Holkeri H. Vitale A. (2001). Vacuolar sorting determinants within a plant storage protein trimer act cumulatively. *Traffic* 2 737–741 10.1034/j.1600-0854.2001.21008.x 11576450

[B23] Ibl V. Stoger E. (2012). The formation, function and fate of protein storage compartments in seeds. *Protoplasma* 249 379–392 10.1007/s00709-011-0288-z 21614590

[B24] Jones A. M. Herman E. M. (1993). KDEL-containing auxin binding protein is secreted to the plasma membrane and cell wall. *Plant Physiol.* 101 595–606 12231715 10.1104/pp.101.2.595 PMC160609

[B25] Kawagoe Y. Suzuki K. Tasaki M. Yasuda H. Akagi K. Katoh E. (2005). The critical role of disulfide bond formation in protein sorting in the endosperm of rice. *Plant Cell* 17 1141–1153 10.1105/tpc.105.030668 15749763 PMC1087992

[B26] Kinney A. J. Jung R. Herman E. M. (2001). Cosuppression of the α subunits of β-conglycinin in transgenic soybean seeds induces the formation of endoplasmic reticulum-derived protein bodies. *Plant Cell* 13 1165–1178 11340189 10.1105/tpc.13.5.1165 PMC135556

[B27] Klein E. M. Mascheroni L. Pomp A. Ragni L. Weimar T. Lilley K. S. (2006). Plant endoplasmin supports the protein secretory pathway and has a role in proliferating tissues. *Plant J.* 48 657–673 10.1111/j.1365-313X.2006.02904.x 17059403

[B28] Kogan M. J. Lopez O. Cocera M. Lopez-Iglesias C. De La Maza A. Giralt E. (2004). Exploring the interaction of the surfactant N-terminal domain of gamma-Zein with soybean phosphatidylcholine liposomes. *Biopolymers* 73 258–268 10.1002/bip.10578 14755582

[B29] Lending C. R. Larkins B. A. (1989). Changes in the zein composition of protein bodies during maize endosperm development. *Plant Cell* 1 1011–1023 10.1105/tpc.1.10.1011 2562552 PMC159838

[B30] Levanony H. Rubin R. Altschuler Y. Galili G. (1992). Evidence or a novel route of wheat storage proteins to vacuoles. *J. Cell Biol.* 119 1117–1128 10.1083/jcb.119.5.1117 1447291 PMC2289714

[B31] Llop-Tous I. Madurga S. Giralt E. Marzabal P. Torrent M. Ludevid M. D. (2010). Relevant elements of a maize γ-zein domain involved in protein body biogenesis. *J. Biol. Chem.* 285 35633–35644 10.1074/jbc.M110.116285 20829359 PMC2975188

[B32] Lombardi A. Marshall R. S. Castellazzi C. L. Ceriotti A. (2012). Redox regulation of glutenin subunit assembly in the plant endoplasmic reticulum. *Plant J.* 72 1015–1026 10.1111/tpj.12020 22966775

[B33] Mainieri D. Rossi M. Archinti M. Bellucci M. De Marchis F. Vavassori S. (2004). Zeolin: a new recombinant storage protein constructed using maize γ-zein and bean phaseolin. *Plant Physiol.* 136 3447–3456 10.1104/pp.104.046409 15502013 PMC527144

[B34] Mellor H. Kimball S. R. Jefferson L. S. (1994). Brefeldin A inhibits protein synthesis through the phosphorylation of the alpha-subunit of eukaryotic initiation factor-2. *FEBS Lett.* 350 143–146 10.1016/0014-5793(94)00756-X 8062914

[B35] Moriguchi R. Matsuoka C. Suyama A. Matsuoka K. (2011). Reduction of plant-specific arabinogalactan-type O-glycosylation by treating tobacco plants with ferrous chelator 2,2 ^′^-dipyridyl. *Biosci. Biotechnol. Biochem.* 75 994–996 10.1271/bbb.100884 21597170

[B36] Napier J. A. Richard G. Turner M. F. P. Shewry P. R. (1997). Trafficking of wheat gluten proteins in transgenic tobacco plants: gamma-gliadin does not contain an endoplasmic reticulum retention signal. *Planta* 203 488–494 10.1007/s004250050218 9421932

[B37] Pedrazzini E. Giovinazzo G. Bielli A. de Virgilio M. Frigerio L. Pesca M. (1997). Protein quality control along the route to the plant vacuole. *Plant Cell* 9 1869–1880 10.1105/tpc.9.10.1869 9368420 PMC157028

[B38] Pedrazzini E. Komarova N. Rentsch D. Vitale A. (2013). Traffic routes and signals for the tonoplast. *Traffic* 14 622–628 10.1111/tra.12051 23356396

[B39] Pompa A. De Marchis F. Vitale A. Arcioni S. Bellucci M. (2010). An engineered C-terminal disulfide bond can partially replace the phaseolin vacuolar sorting signal. *Plant J.* 61 782–791 10.1111/j.1365-313X.2009.04113.x 20030752

[B40] Pompa A. Vitale A. (2006). Retention of a bean phaseolin/maize gamma-zein fusion in the endoplasmic reticulum depends on disulfide bond formation. *Plant Cell* 18 2608–2621 10.1105/tpc.106.042226 17041149 PMC1626613

[B41] Prat S. Cortadas J. Puigdomenech P. Palau J. (1985). Nucleic acid (cDNA) and amino acid sequences of the maize endosperm protein glutelin-2. *Nucleic Acids Res.* 13 1493–1504 10.1093/nar/13.5.1493 3839076 PMC341091

[B42] Reyes F. C. Chung T. Holding D. Jung R. Vierstra R. Otegui M. S. (2011). Delivery of prolamins to the protein storage vacuole in maize aleurone cells. *Plant Cell* 23 769–784 10.1105/tpc.110.082156 21343414 PMC3077793

[B43] Shewry P. R. Napier J. A. Tatham A. S. (1995). Seed storage proteins: structures and biosynthesis. *Plant Cell* 7 945–956 10.1105/tpc.7.7.945 7640527 PMC160892

[B44] Tabe L. M. Wardley-Richardson T. Ceriotti A. Aryan A. McNabb W. Moore A. (1995). A biotechnological approach to improving the nutritive value of alfalfa. *J. Anim. Sci.* 73 2752–2759 8582868 10.2527/1995.7392752x

[B45] Torrent M. Llompart B. Lasserre-Ramassamy S. Llop-Tous I. Bastida M. Marzabal P. (2009). Eukaryotic protein production in designed storage organelles. *BMC Biol.* 7:5 10.1186/1741-7007-7-5 PMC2637842 19175916

[B46] Virgili-López G. Langhans M. Bubeck J. Pedrazzini E. Gouzerh G. Neuhaus J.-M. (2013). Comparison of membrane targeting strategies for the accumulation of the human immunodeficiency virus p24 protein in transgenic tobacco. *Int. J. Mol. Sci.* 14 13241–13265 10.3390/ijms140713241 23803657 PMC3742185

[B47] Vitale A. Hinz G. (2005). Sorting of proteins to storage vacuoles: how many mechanisms? *Trends Plant Sci.* 10 316–323 10.1016/j.tplants.2005.05.001 15950520

[B48] Vitale A. Smaniotto E. Longhi R. Galante E. (1982). Reduced soluble proteins associated with maize endosperm protein bodies. *J. Exp. Bot.* 33 439–448 10.1093/jxb/33.3.439

[B49] Wang G. Wang F. Wang G. Wang F. Zhang X. Zhong M. (2012). Opaque1 encodes a myosin XI motor protein that is required for endoplasmic reticulum motility and protein body formation in maize endosperm. *Plant Cell* 24 3447–3462 10.1105/tpc.112.101360 22892319 PMC3462643

[B50] Woo Y.-M. Hu D. W.-N. Larkins B. A. Jung R. (2001). Genomics analysis of genes expressed in maize endosperm identifies novel seed proteins and clarifies patterns of zein gene expression. *Plant Cell* 13 2297–2317 10.1105/tpc.13.10.2297 11595803 PMC139160

[B51] Wu Y. Messing J. (2010). RNA interference-mediated change in protein body morphology and seed opacity through loss of different zein proteins. *Plant Physiol.* 153 337–347 10.1104/pp.110.154690 20237020 PMC2862413

[B52] Xu J. H. Messing J. (2008). Organization of the prolamin gene family provides insight into the evolution of the maize genome and gene duplications in grass species. *Proc. Natl. Acad. Sci. U.S.A.* 105 14330–14335 10.1073/pnas.0807026105 18794528 PMC2567223

[B53] Xu J. H. Messing J. (2009). Amplification of prolamin storage protein genes in different subfamilies of the Poaceae. *Theor. Appl. Genet.* 119 1397–1412 10.1007/s00122-009-1143-x 19727653

[B54] Yuan L. Dou Y. Kianian S. F. Zhang C. Holding D. R. (2014). Deletion mutagenesis identifies a haploinsufficient role for γ-zein in opaque2 endosperm modification. *Plant Physiol.* 164 119–130 10.1104/pp.113.230961 24214534 PMC3875793

